# Identification of a balanced complex chromosomal rearrangement involving chromosomes 3, 18 and 21 with recurrent abortion: case report

**DOI:** 10.1186/1755-8166-7-39

**Published:** 2014-06-05

**Authors:** Yaping Liao, Liqun Wang, Ding Zhang, Changqing Liu

**Affiliations:** 1 Department of Cell Biology, Bengbu Medical College, Bengbu 233030, China; 2 Department of obstetrics and gynecology, the First Affiliated Hospital, Bengbu Medical College, Bengbu, Anhui, People’s Republic of China

**Keywords:** Complex chromosomal rearrangements (CCRs), Recurrent spontaneous abortions, Genetic counseling, Fluorescence in situ hybridization

## Abstract

**Background:**

Complex chromosome rearrangements (CCRs) are constitutional structural rearrangements involve more than two breakpoints on two or more chromosomes. Balanced CCR carriers are often phenotypically normal but associated with high risk of spontaneous abortion and having abnormal offspring with unbalanced karyotype. Here, we report a new familial case of complex chromosome structural aberrations involving chromosomes 3, 18 and 21 and four breakpoints.

**Results:**

Cytogenetic investigations showed a complex chromosomal chromosome rearrangement involving chromosomes 3, 18 and 21 with four breakpoints. 2 of 4 breakpoints were within the long arm of chromosome 18. Three-color fluorescence in situ hybridization (FISH) confirmed the complexity of the rearrangement and showed the derivative 21 to be composed of 3 distinct segments derived from chromosomes 21, 18, and 3. The karyotype of CCR carrier was determined as 46,XX,t(3;21;18)(3pter → 3q12::18q23 → 18qter;21pter → 21q22.1::18q21.1 → 18q23::3q12 → 3qter; 18pter → 18q21.1::21q22.1 → 21qter).

**Discussion:**

A new complex balanced CCR was characterized using conventional high resolution banding and molecular cytogenetic analysis. The results provided an explanation of recurrent abortion and abnormal child for balanced CCR carriers. Genetic counselling and prenatal diagnosis for couples with a balanced CCR is necessary since they have a high risk of having a child with unbalanced karyotype. Additional studies to reveal the molecular mechanism of CCRs would help reveal the rule of inherited CCRs in offspring.

## Background

Complex chromosome rearrangements (CCRs) are structural aberrations involving at least three breakpoints on two or more chromosomes and exchange of genetic material between these chromosomes. Translocation, insertion and transposition are often involved in CCRs. CCRs are rare in humans and can be familial or de novo [1,2]. So far, ~255 cases of CCRs involving three or more chromosomes have been reported and most are de novo [3].

It has been observed that most balanced CCRs occur in females, and about half of them are inherited [4]. In males, balanced CCRs are often subfertile or sterile due to spermatogenesis disturbance [2,3,5,6]. Although balanced CCR carriers are not often associated with abnormal phenotypes, a high risk of miscarriage and live born child with an unbalanced karyotype are found. It is difficult to identify CCRs correctly by conventional cytogenetics based on banding techniques without the aid of additional diagnostic tools such as fluorescence in situ hybridization (FISH) or other advanced molecular cytogenetic techniques [7-10].

Here, we present a family with at least three unbalanced or balanced CCR carriers involving chromosomes 3, 18 and 21 using traditional high resolution banding and three-color FISH.

## Case presentation

**Figure 1:**
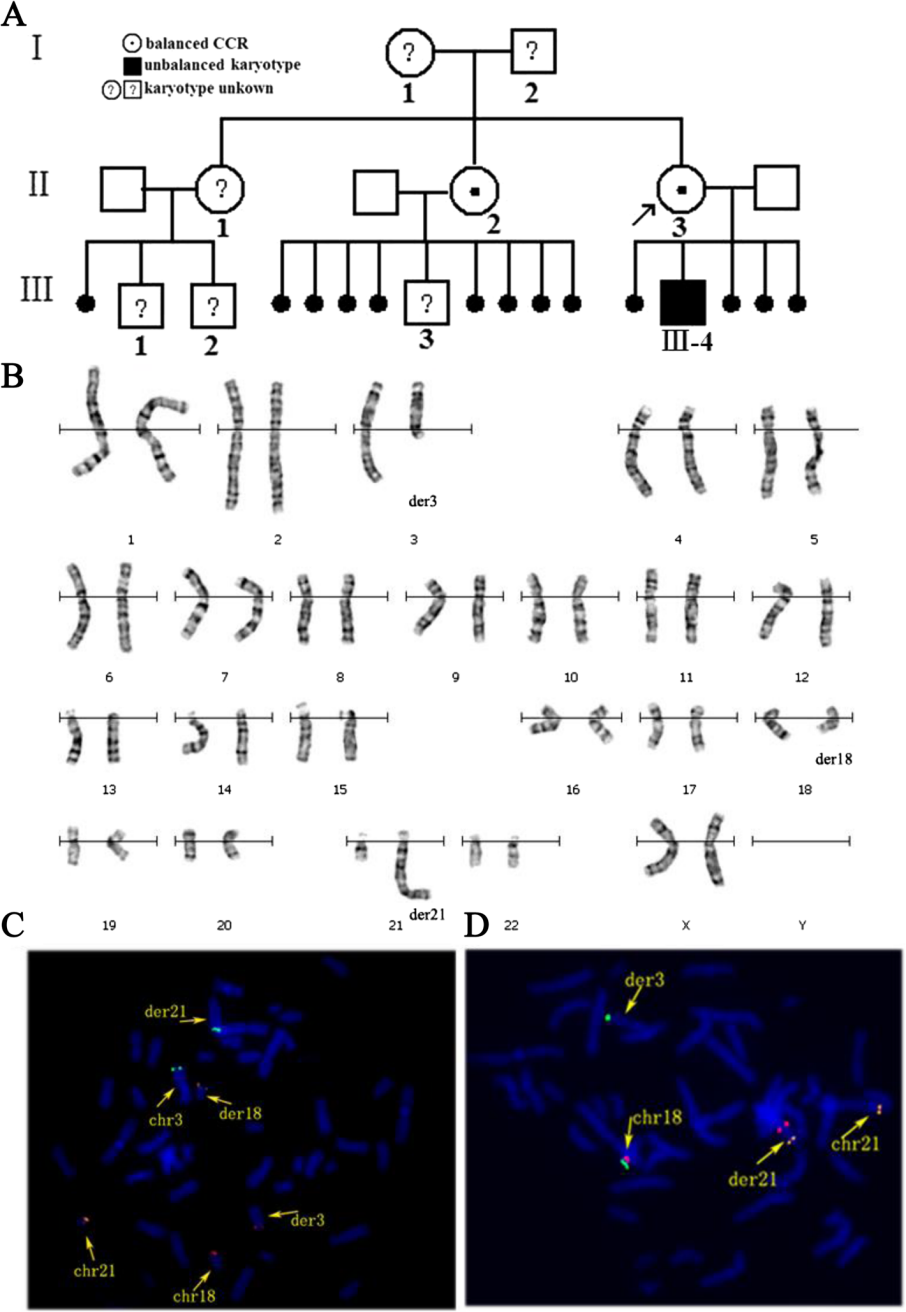
**Identification of a complex translocation involving chromosomes 3, 18 and 21. A**: Pedigree of the proband’s family (arrow). **B**: GTG banded karyotype of the proband showing three derivative chromosomes. **C**: BAC-probes RP11-379C23 (green) (3q27. 2), RP11-190A24 (21q22.3) (orange) and RP11-89 N1 (red) (18q23) demonstrate a translocation among chromosomes 3, 21 and 18. **D**: BAC-probes TRP11-7H17 (18q23) (green), BAC-probe RP11-57 F7 (18q22.2) (red) and RP11-89H21(21q11.2) (orange) show the insertion of part of chromosome 18 in derivative chromosome 21.

The proband (II-3), a 31-year old woman, and her husband were referred by cytogenetic investigation because the proband had four first-trimester miscarriages (Figure 1A). The physical examination revealed that the proband (II-3) and her husband were phenotypically normal including their reproductive systems. The proband had a 7-year old son (III-4) with typical symptoms and physical characteristics of Down syndrome including mental retardation and physical growth delay [11,12]. He was born at 39 weeks of gestation with a weight of 3.1 kg and a length of 47 cm. He had a height of 103 cm and a weight of 18 kg at 7-year old.

The proband (II-3) had two sisters. The eldest sister, 43-year old, had one miscarriage (~22 weeks) and two phenotypically normal boys. The second elder sister (II-2), 40-year old, had eight first-trimester miscarriages and one 12-year old boy who had normal phenotype and normal or balanced karyotype. Recurrent abortion at first-trimester and one abnormal child occurred in this family suggested a possible chromosomal aberration.

## Results

The blood karyotype from the proband (II-3) revealed a translocation involving chromosomes 3, 21 and 18 (Figure 1B). Additionally, it seems that a segment from 18q21 ~ q23 inserted to der 21(q22) when high resolution staining karyotype analysis was used, but it could not be karyotypically determined. The husband had a normal karyotype both by GTG banding and high resolution staining (data not shown). Analysis of the siblings revealed different cytogenetic anomalies. A sister (II-2) showed the same chromosome rearrangement as that of the proband (II-3), whereas the son (III-4) of the proband had unbalanced karyotype carrying not only the der (3), der (18) and der (21) but also two normal chromosome 21. So he was diagnosed as Down syndrome according to karyotype and typical symptoms.

To identify complex chromosomal rearrangements or subtle translocations, three-color FISH was performed with regional specific BAC-probes (Table 1) as described previously [13]. The BAC-probe RP11-379C23 (3q27. 2), RP11-190A24 (21q22.3) and RP11-89 N1 (18q23) located near to telomeres confirmed a translocation between chromosomes 3, 21 and 18 (Figure 1C). The BAC-probe TRP11-7H17 (18q23) was used as a control, and it showed hybridization to the der (3) (Figure 1D). However, the BAC-probe RP11-57 F7 (18q22.2) was used as a second control, it showed hybridization to the der (21) (Figure 1D). Based on FISH and cytogenetic results, there are two breakpoints located in abnormal chromosome 18q. One breakpoint was located in 18q22.2 ~ 18q23, and the other one was located in 18q21.1. The middle fragment inserted in der (21q22) was derived from chromosome 18. Thus, the present case is not a simple three-way CCR and the karyotype was readjusted and assigned according to ISCN 2013 as follows: 46,XX,t(3;21;18)(3pter → 3q12::18q23 → 18qter;21pter → 21q22.1::18q21.1 → 18q23::3q12 → 3qter; 18pter → 18q21.1::21q22.1 → 21qter).

**Table 1:** Fluorescence in situ hybridization analysis with the following probes used

**Clone name**	**Location**	**Mb-position**	**Color labeled**	**Result**
RP11-379C23	3q27. 2	186,968 bp	Spectrum Green	3, der21
RP11-89 N1	18q23	187,919 bp	Spectrum Red	18, der3
RP11-7H17	18q23	185,880 bp	Spectrum Green	18, der3
RP11-57 F7	18q22.2	158,051 bp	Spectrum Red	18, der21
RP11-190A24	21q22.3	171,318 bp	Spectrum Orange	21, der18
RP11-89H21	21q11.2	515 bp	Spectrum Orange	21, der21

## Discussion

Although CCRs are rarely found in screened populations, they are often associated with mental retardation, congenital abnormalities, recurrent spontaneous abortions and infertility [14-16]. The application of FISH and its derivative techniques facilitated the characterization of CCRs and become essential for further delineation of chromosomal breakpoints [3,17]. The aim of this study was to identify whether a complex abnormal karyotype was apparent or not, and whether possible breakpoints were involved in chromosomes using high-resolution chromosome analysis and FISH. This is especially important for prenatal diagnosis and appropriate genetic counseling [18].

In the present case, the proband (II-3) was found to carry balanced CCRs involving chromosomes 3, 18 and 21 with four breakpoints. The four breakpoints were located in 3q12, 18q21.1, 18q23 and 21q22.1. To the best of our knowledge, this is the first report of a balanced CCR involving chromosomes 3, 18 and 21 with 2 breakpoints on chromosome 18. By combining FISH and high resolution banding data, we changed the previous description of this CCR from a simple three-way translocation to the extra complex CCR according to the category of CCRs proposed by Kausch et al. [19] and Madan [4]. In this family, we proposed that one of the proband’s parent was carrying the balanced CCR since the complete balanced CCR was present in at least two of their offspring: II-2 and II-3 (Figure 1A). It was previously observed that transmission of CCR was usually maternal since male carriers have an increased risk of primary infertility or subfertility [16]. We inferred the familial CCRs possibly inherited from her mother. However, this hypothesis could not be confirmed because her parent’s karyotype could not be studied due to the unavailability of blood samples.

**Figure 2:**
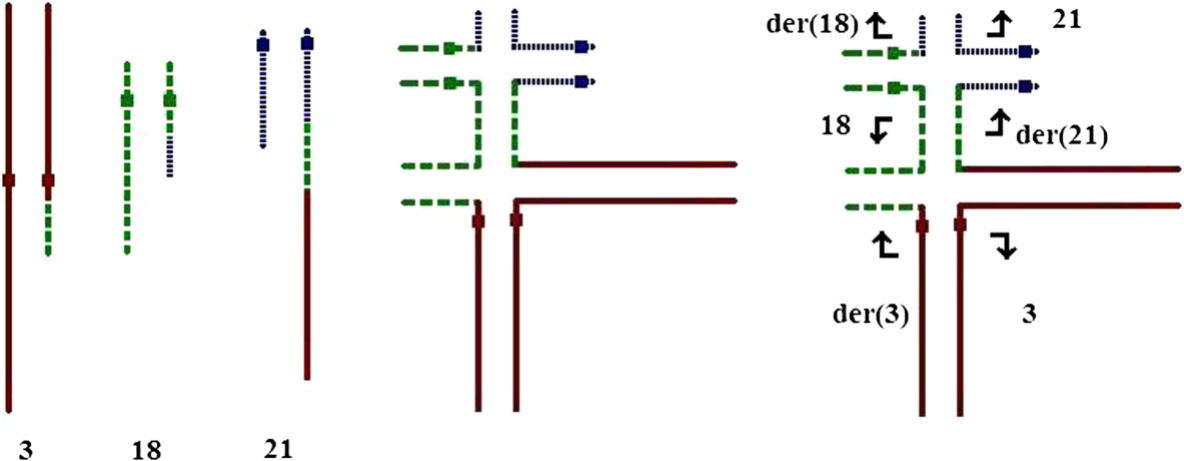
**Theoretical pachytene configuration.** Pachytene diagram of the proband (II-3) and segregation mode of her son (III-4). The arrows indicate the direction of separation to each pole.

It is difficult to obtain the exact rate of meiotic segregation from the ovum of balanced CCR carriers because most of ovum cannot be obtained. In an attempt to understand the mechanisms of meiotic segregation, Loup et al. [20] analyzed meiotic segregation in the sperm of a patient with a three-way familial CCR. They found a high rate of unbalanced sperm (75.9%) including 34.1% from 3:3 segregation, 38.2% from 4:2 segregation, 3.5% from 5:1 segregation and 0.05% from 6:0 segregation. Only 14.8% of sperm were normal or balanced. We think the mode of meiotic segregation may be various in different type of CCRs. Recently, Madan [4] reviewed the mode of segregation of 68 offspring of 63 CCR carriers. They found a clear difference between the simple type of CCR (three-way or four-way CCR) where 4:2 segregation was more frequent (14/26) and the extra complex CCRs (number of breaks > number of chromosomes) where adjacent 1 segregation took place in the majority of cases (39/42).In the present case with extra complex CCR, there are several possibilities for a high incidence of abnormal pregnancy outcome. At the pachytene stage, the CCR will form a possible hexavalent configuration different from three-way translocation (Figure 2). Generally, unbalanced 3:3, 4:2, 5:1 and 6:0 segregations often produce greater genomic imbalance, and early pregnancy loss are expected.. In addition, recombination in the inserted segment can result in gametes with new unbalanced karyotype. However, live born abnormal child with Down syndrome is possible though 4:2 segregation because chromosome 21 is involved in this CCR. The proband (II-3) had a son (III-4) who had unbalanced CCR with abnormal phynotype. According to G-banding karyotype, he gained the der (3), der(18), der(21) and normal chromosome 21 from his mother (Figure 2). Therefore, we confirmed that the mode of meiotic segregation is 4:2 in our patient.

Genetic counseling for CCR carriers is very important and can be offered before and after pregnancy. Madan et al. [16] reviewed 60 familial and de novo cases of balanced CCRs, and estimated that carriers have a 50% risk of spontaneous abortion and a 20% risk of having a child with an unbalanced karyotype. Although published data are a good rough guide for counseling, we think it cannot be applied to individual CCRs as the risk estimates remains highly empirical. The category of CCRs and the number of chromosomes involved can vary greatly giving a wide variety of possible gametes [19,20]. In our case, 4/5 pregnancies ended in a spontaneous abortion and a child with unbalanced karyotype (1/5). One of the proband’s sister (II-2) with the same karoytpe had high frequent spontaneous abortion (8/9) and one phenotype normal child (1/9). A total of 12/14 pregnancies from two carriers ended in spontaneous abortion indicated an increased risk of miscarriage in this family higher than previously reported [16,21]. Considering the high incidence of abnormal pregnancies, natural pregnancy of the balanced carriers is not encouraged in this family. Even if the balanced carriers conceive a child naturally, prenatal diagnosis for balanced CCR carriers is necessary owing to an estimated risk of 7.1% (1/14) having a child with Down syndrome in this family. It has been thought preimplantation genetic diagnosis (PGD) is impossible for CCRs carries as the highly complexity of meiotic segregation. Recently, several studies reported that healthy live birth after successful PGD of CCR carries. These data indicate that CCRs are amenable to PGD analysis as well as egg donation, after a proper genetic counselling [22-24].

## Conclusion

We report here a familial case of CCRs possibly inherited from her mother. The balanced CCRs resulted in recurrent abortion and an abnormal child with unbalanced karyotype. It is very important to identify the chromosomes and the breakpoints involved in CCRs as accurately as possible to understand the mechanism underlying the formation of CCRs and to provide correlation between phenotype and chromosomal aberration. With accurate characterization of CCRs, correct prenatal diagnosis and efficient genetic counseling can be made. Our data indicate that the extra complex CCRs have higher incidence of abnormal pregnancy outcome, and that it is difficult to predict the exact risk of having a child with unbalanced karyotype. Additional studies to reveal the molecular mechanism of CCRs would help reveal the rule of inherited CCRs in offspring.

## Materials and methods

### Karyotyping

Cytogenetic analysis was performed on peripheral blood cultures after 72 h of incubation. Metaphase spreads were prepared for GTG banding and high-resolution staining according to standard procedures. Karyotypes were obtained from the proband, her husband, her son, and her second elder sister. The proband’s parent (I-1 and I-2) and three children (III-1, III-2 and III-3) of proband’s sisters could not be studied due to the unavailability of blood samples from these members. Twenty metaphases were analyzed from each subject by GTG banding. Olympus microscope (BX41) was used for karyotyping and metaphase images were captured using VideoTesT-Karyo software (Metasystems, Altlussheim, Germany).

### FISH

To identify balanced CCR or subtle translocation, three-color FISH analysis was performed for the proband following conventional protocols as previously described [13]. Specific bacterial artificial chromosome (BAC) clones were selected from the human library RPCI-11 according to the UCSC Human Genome Assembly (UC Santa Cruz, USA, assembly February 2009) and provided by State Key Laboratory of Medical Genetics, Central South University. BAC DNA was directly labeled with spectrum Green, spectrum Orange and Spectrum Red-dUTP using nick translation (Table 1). The chromosomes were made fluorescent by 4′, 6-diamidino-2-phenylindole (DAPI). Each of the 20 metaphase spreads was analyzed using a fluorescence microscope (Leica DM5000B). Images were captured and processed by using Leica CW 4000 software.

## Consent

Written informed consent was obtained from the patient for publication of this Case report and any accompanying images.

## Abbreviations

CCRs: Complex chromosomal rearrangements; FISH: Fluorescence in situ hybridization; BAC: Bacterial artificial chromosome; PGD: Preimplantation genetic diagnosis.

## Competing interests

The authors declare that they have no competing interests.

## Authors’ contributions

LP and LQ drafted the paper and participated in the molecular cytogenetic analysis. WQ evaluated the family with examination and genetic counseling. ZD did the cytogenetic analysis. All authors read and approved the final manuscript.
